# Implementation of a heart failure readmission reduction program: a role for medical residents

**DOI:** 10.3402/jchimp.v2i1.10674

**Published:** 2012-04-30

**Authors:** Jennifer Rabbat, Daniel R. Bashari, Rajnish Khillan, Manisha Rai, Jose Villamil, Julie M. Pearson, Archana Saxena

**Affiliations:** 1 Department of Internal Medicine, Lutheran Medical Center, Brooklyn, NY, USA; 2 Department of Clinical Research, Lutheran Medical Center, Brooklyn, NY, USA; 3 Department of Cardiology, Lutheran Medical Center, Brooklyn, NY, USA

**Keywords:** Heart failure, hospital readmission, medical residency, documentation, quality improvement, systems-based practice

## Abstract

**Background:**

Congestive heart failure (CHF) is one of the leading causes of hospital readmissions within 30 days of discharge. Due to the substantial costs associated with these readmissions, several interventions to reduce CHF readmissions have been developed and implemented.

**Methods:**

To reduce CHF readmissions at our community teaching hospital, the Smooth Transitions Equal Less Readmission (STELR) program was developed. Utilizing the Plan-Do-Check-Act cycle for quality improvement, resident physicians tracked patients enrolled in the STELR program. The resident contribution to the program was substantial in that they were able to quantify the improvement in both physician practices and patient readmissions. This provided insight into program areas requiring further modification, which the hospital would not have obtained without resident participation.

**Results:**

The readmission rate for patients diagnosed with heart failure decreased from 32% prior to program implementation, to 24% hospital wide (including patients who were not tracked in the STELR program), and 21% among patients tracked by the residents.

**Conclusion:**

This effective CHF readmission reduction program requires less financial resources compared to government funded programs. The resident involvement in the STELR program helped to assess and improve the program and also allowed the residents to gain an awareness of the resources available to their patients to facilitate their transition home. The program exposed the residents to systems-based practice, a fundamental element of their residency training and, more generally, community care.

Congestive heart failure (CHF) is the most common indication for hospitalization due to exacerbation of a chronic condition among adults aged 65 years and older in the United States (1). Heart failure related hospitalizations have increased from 1,274,000 in 1979 to 3,860,000 in 2004 (2). The annual cost associated with caring for heart failure patients is estimated at nearly 20 billion dollars (3) and is primarily attributed to frequent hospital readmissions due to decompensation (4). Factors associated with readmission due to an exacerbation of CHF symptoms include advanced age, prior hospital admission, length of hospital stay, severity of illness, and medical comorbidities (4–7).

Several interventions to reduce CHF readmissions have been developed and implemented. One intervention in the outpatient setting utilizes home health care nurses to provide disease and treatment education, identify and manage early decompensations and bolster family support (8–17). Better Outcomes for Older adults through Safe Transitions (Project BOOST) is a multidisciplinary program to facilitate communication between inpatient and outpatient settings. Data-driven resources and expert mentoring are provided to optimize patient discharge, improve communication between inpatient and outpatient providers and educate patients and families (18). Preliminary findings demonstrate a reduction in 30-day readmission rates from 14.2 to 11.2% within 6 months of Project BOOST implementation (19). Another intervention, Re-Engineered Discharge (Project RED), utilizes a nurse discharge advocate to help patients understand after-hospital care instructions, such as how to take their medicines and when to make follow-up appointments (20, 21). When patients understand this better, they are 30% less likely to be readmitted or visit the emergency department (22).

To reduce CHF readmissions at our community hospital, a committee was formed with hospital administration, the Chief of Cardiology and other attending physicians, nurse care coordinators, documentation specialists, and case managers who developed Smooth Transitions Equals Less Readmissions (STELR). The aims of the STELR program were to improve medical staff diagnosis, management, and treatment of heart failure, based on current standards of care. It also sought to facilitate smooth patient transitions to home by providing education regarding their condition during their hospitalization, and by setting up home health services and a follow-up appointment within 7 days of discharge. To maximize limited resources at our community teaching hospital, resident physicians tracked patients enrolled in the STELR program contributing to the implementation. The program also provided residents an opportunity for peer-to-peer education and learning about systems-based practice, a core competency requirement of the Accreditation Council for Graduate Medical Education (ACGME).

The purpose of this paper is to describe the integration of residents into the implementation of a CHF readmission reduction program at a community hospital and assess the residents’ contribution to the program. Utilizing the Plan-Do-Check-Act cycle (23, 24) for quality improvement, the residents assisted the check phase of the STELR program. The Plan-Do-Check-Act cycle is used to test the potential effects of a quality intervention on processes, leading to a larger and more targeted change. During the check phase, interventional processes are measured and results are compared to baseline findings (23). Accordingly, the residents collected and analyzed data to monitor the improvements during the pilot test, which is described here. An assessment of the STELR program in achieving the overall goal of reducing readmissions among patients with CHF is also provided.

## Methods

STELR was developed at Lutheran Medical Center, a community teaching hospital in Sunset Park, Brooklyn. The 476 bed medical center serves a diverse, multicultural, largely immigrant population. Data related to the STELR program included a retrospective and prospective component, which is described in detail below.

### Retrospective data collection

Retrospective data were collected to characterize CHF patients who were readmitted within 30 days of discharge at our hospital and to identify potential areas for intervention. Information Technology specialists derived a list of patients with a primary discharge diagnosis of CHF who were readmitted within 30 days of discharge, from November 2009 to April 2010. The CHF readmission rate prior to STELR was also calculated. Readmission rates were calculated as the percentage of CHF patients who were readmitted to the hospital within 30 days of discharge for any cause (25). Data were extracted from the index admission on 65 patients who were readmitted within 30 days after discharge for the components of the STELR program.

### Prospective data collection

Prospective data were collected to evaluate the STELR program overall by tracking patients who were enrolled in the intervention. Patient data in this component represents both patients who were and were not readmitted within 30 days of discharge (therefore, statistical conclusions between the patient characteristics of the groups is unwarranted).

Patients were initially identified by using a list of patients admitted from November 2010 through January 2011 to the telemetry unit who had shortness of breath, CHF, or edema, documented as a working diagnosis upon admission. Based on this list, patients were screened with a heart failure diagnostic tool. The CHF diagnostic tool included:

1. Symptoms: dyspnea, orthopnea, fatigue worse than baseline, lower extremity edema, paroxysmal nocturnal dyspnea, weight gain, and change in mentation.
2. Behaviors: non-compliance with medications and excessive sodium intake.
3. Physical exam findings: crackles, elevated JVD, LE edema, S3 gallop.
4. Diagnostic tests consistent with CHF: 2DEcho, B-type natriuretic peptide (BNP) MUGA, cardiac catheterization.

Patients with advanced directives for hospice care were excluded from the study prior to undergoing any screening.

Patients who screened positive for CHF were tracked from admission to discharge. In addition, patients’ electronic medical records were monitored for 30 days post-discharge to determine if a readmission occurred. A readmission was determined by viewing visit histories through the electronic medical record. Residents on the study team enrolled and tracked 69 patients in the STELR program. There were additional patients who received the STELR program's interventions during the specified time period; however, some of these patients were not tracked by the residents if they did not have one of the specific working diagnoses, or if they were admitted and transferred off the telemetry unit at night or over a weekend.

Patients younger than 18 years old were excluded from both the retrospective and prospective components of the study. The study was approved by the hospital's Institutional Review Board.

### Data analysis

All study data were entered by the participating residents into a Microsoft Excel spreadsheet. Descriptive statistics were calculated using SPSS software (V.18).

### The STELR program

The five residents on the STELR team (co-authors, J.R., D.R.B., R.K., M.R., and J.V.) collaborated with other STELR team members to carry out program components such as peer-to-peer education, nurse-driven patient education, and ensuring patient follow up after discharge. During this time the residents took turns overseeing program implementation and spent approximately 10 h per week implementing the program and 1 h per week meeting with the entire research team. A large component of the resident STELR team participation involved tracking patients through the program to help monitor program implementation. The resident STELR team completed these tasks in addition to their regular clinical responsibilities. The patients were cared for by the primary team on the floor; however, the resident STELR team provided indirect care for the patients with respect to the peer-to-peer education of junior residents, so they could learn more effective and aggressive management of heart failure patients. In addition, the residents consulted with nursing staff to ensure heart failure teaching was completed and helped junior residents with scheduling processes to ensure swift patient follow up.

#### Peer-to-peer education

Residents reviewed STELR patient charts for accurate documentation of the clinical and laboratory data for heart failure as indicated on the diagnostic tool. Evidence-based core competency measures for heart failure patients were taught and reinforced to the residents managing that particular patient by the research team and documentation specialist. Residents also advised their peers on issues such as identifying the severity of the disease process, uptitrating medications, adding additional CHF therapies, as well as formulating and documenting a clear treatment plan. The charts were also examined to see if notation was made of the type and stage of heart failure, and the New York Heart Association (NYHA) class of the patient upon admission.

The resident STELR team encouraged their peers to follow strict standard of care measures for patients with heart failure including the daily fluid balance, daily weights, and routine blood tests including electrolytes. Physicians on the committee emphasized the importance of medication reconciliation with home medications, and of prescribing generics and medications available through $4 prescription programs to foster patient compliance with medications. The resident STELR team mentored other resident physicians with respect to these measures when appropriate and assisted with the medication reconciliation form for each patient upon discharge, when needed.

#### Patient education

A fundamental aspect of the program was nurse-driven patient-centered education with distribution of heart failure management manuals. Licensed registered nurses educated patients on heart failure after participating in a heart failure in-service training by senior nursing staff members. Symptom recognition and warning signs of heart failure exacerbation were emphasized through written and audiovisual materials. The importance of behavior modification such as dietary and medication compliance was reinforced by giving written handouts regarding patient's medication regimen, along with a pillbox and dietary information. The research team residents documented which patients received heart failure education on the prospective data collection form. If CHF education was not provided, the resident STELR team instructed the nurses to complete the education at the next available opportunity.

#### Ensuring follow up

The residents on the STELR team ensured that the primary resident for the patient scheduled a follow-up appointment within 7 days of discharge with the patient's private internist or cardiologist. For those patients without primary care physicians, an appointment was made for them in the general medicine, cardiology, or heart failure clinic. Residents were asked to write this information down on the discharge instruction form. Clerical staff members were trained to schedule outpatient appointments via the computerized outpatient scheduling system 24 h a day, 7 days a week. When possible, the STELR resident team checked that the clerks scheduled these appointments and provided printouts with the details for the patient prior to discharge. In addition to aiding the scheduling of follow-up appointments, the resident STELR team collaborated with case managers to assess the need for home health care services. Case management worked with onsite home health care staff to set-up home health care services prior to discharge. Finally, a medication reconciliation form was completed for each CHF patient discharged by the primary resident.

## Results

### Retrospective

Sixty-five patients were readmitted within 30 days of initial discharge between November 2009 and April 2010. The index admission chart showed that these patients were between the ages of 49 and 99 years old (average of 77±10 years). Fifty-one percent of the patients were male and 86% of patients had Medicare as their primary form of insurance. Forty percent of the readmitted patients were diagnosed with systolic heart failure, while 37% were diagnosed as having heart failure with preserved systolic function. The remainder of the patients had right sided heart failure or combined heart failure. The majority of patients had hypertension (75%) and coronary heart disease (60%) in addition to CHF (Table 1).

Regarding discharge, 32% of patients were referred to a home health care agency prior to discharge, while 42% of patients had a follow-up appointment scheduled within 7 days of hospital discharge. Twenty-two percent of all readmitted patients were initially admitted to the hospital from a long-term skilled nursing facility while 31% of patients were discharged to a long-term skilled nursing facility prior to their readmission. All 65 patients were discharged on 8 or more medications (average of 10 medications).

Several deficiencies in documentation were noted. For instance, 97% of the patient charts that were reviewed did not document the NYHA Classification, weight gain/loss, or excessive sodium intake (Table 2). In addition, patient care monitoring was insufficient; only 31% of readmitted patients had daily weights ordered on their admission.

### Prospective

Sixty-nine patients were tracked by the STELR resident team. The age range of all prospectively tracked patients was between 54 and 102 (average 79±12 years) and 52% of the patients were female and 48% were male. Medicare was the most common primary insurance provider (78%). Six patients expired during the course of the study. The majority of patients were diagnosed with systolic heart failure (48%), followed by heart failure with preserved systolic function (29%). Right-sided heart failure and combined heart failure accounted for the remainder of the patients. Readmitted patients in the retrospective component were not sicker than patients in the prospective component even though fewer patients who were tracked during STELR were readmitted (Table 1).

**Table 1:** Patient characteristics

Co-morbidity	Retrospective *n* (%)	Prospective *n* (%)
Hypertension	49 (75)	59 (86)
Chronic artery disease	39 (60)	44 (64)
Diabetes	30 (46)	24 (35)
Atrial fibrillation	25 (39)	27 (39)
Smoking (current or former)	21 (32)	35 (51)
ESRD on hemodialysis	16 (25)	7 (10)
Chronic pulmonary disease	13 (20)	17 (25)
Stroke	5 (8)	6 (9)

#### Peer-to-peer education

Ninety percent of the primary residents who had STELR patients assigned to them received education from the resident research team about correct medications, uptitrating doses, core measures, admission criteria, and correct coding and documentation. There was a marked improvement in resident physician documentation for pertinent positive and negative findings in the charts of patients who were tracked in the STELR program. Improvement was noted in documentation of NYHA Classification, weight gain, orthopnea, non-compliance with medications, excessive sodium intake and paroxysmal nocturnal dyspnea (PND) (Table 2). In addition, patient care monitoring improved during the STELR program. Among the tracked patients, 78% had daily weights ordered.

**Table 2:** Percentage of patient charts with documentation for pertinent positive and negative findings

	Documentation	Retrospective *n* (%)	Prospective *n* (%)
HPI and ROS	Dyspnea	64 (98)	64 (93)
	Subjective lower extremity edema	61 (94)	50 (72)
	Fatigue worse than baseline	39 (60)	47 (68)^a^
	Orthopnea	35 (54)	45 (65)^a^
	PND	26 (40)	35 (51)^a^
	NYHA classification	2 (3)	20 (29)^a^
	Weight gain/loss	2 (3)	18 (26)^a^
	Change in mentation	62 (95)	31 (45)
	Non-compliance with medications	13 (20)	30 (44)^a^
	Excessive sodium intake	2 (3)	21 (30)^a^
Exam	Elevated JVP	47 (72)	38 (56)
	Ascites	46 (71)	28 (41)
	Volume overload	61 (94)	44 (64)
	S3 gallop	23 (35)	21 (30)

a Improved documentation after the STELR program exhibited by an increase in percentage of proper documentation.

#### Patient education

In total, 67% of patients tracked in the STELR program received education regarding their diagnosis and medications from the registered nurses. Of the patients for whom data from the time of discharge was available, 88% received information on their medications and 76% knew their diagnosis at time of discharge. All 69 patients had more than 8 medications prescribed to them at the time of discharge (average of 9 medications).

#### Ensuring follow up

Fifty-four percent of STELR patients had a follow-up appointment within 7 days of hospital discharge written on their discharge instructions, while 39% of appointments made were with the patient's primary care physician. The majority (55%) were discharged home with family support and 13% of the patients were discharged to a long-term skilled nursing facility. Of the 11 STELR tracked patients who were readmitted within 30 days of discharge, 18% were discharged to long-term skilled nursing facilities. Fifty-six percent of STELR patients were referred to a home health care agency prior to their hospital discharge.

### Readmission rates

**Fig. 1:**
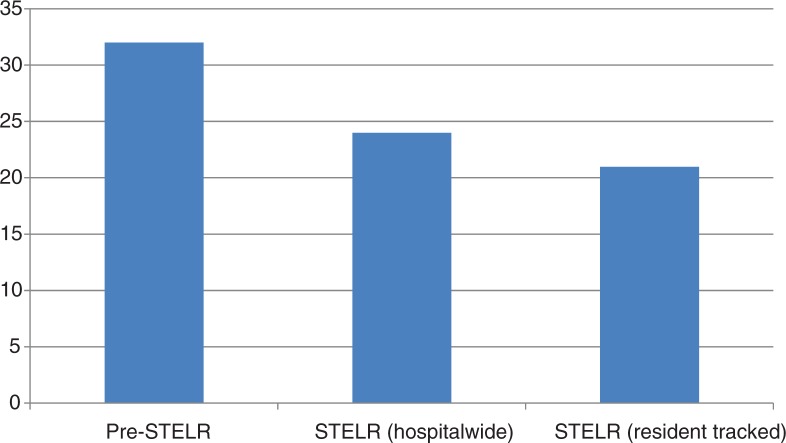
Percentage of CHF patients readmitted within 30 days of discharge.

The readmission rate for patients diagnosed with heart failure admitted during the retrospective data collection period and prior to STELR implementation was 32%. The readmission rate for the entire heart failure population admitted to the hospital during the implementation of STELR, including patients who were not tracked in the STELR program, was 24%. The readmission rate among patients tracked by the residents through the STELR program during the same time period was 21% (Fig. 1).

## Discussion

Resident involvement in the STELR program helped provide insight into program areas requiring modification that would not have been possible without patient tracking. Residents were able to quantify changes in both physician practices and patient readmissions. Patient tracking was advantageous to the overall goal of the STELR program, as indicated by the reduction in CHF readmissions. The readmission rate among patients enrolled in the STELR program (21%) was lower than the hospital wide rate during the same time period (24%). Our data suggests that resident patient tracking had an impact on the success of this transition of care program.

There were marked improvements in several program areas, including physician documentation of correct diagnoses, and pertinent signs and symptoms of heart failure. Improved documentation leads to a more organized thought process for the physician and team involved in patient care. In addition, due to resident work hour restrictions and an increase in patient hand-offs, clear documentation improves patient outcomes as progression and deterioration can be easily assessed over the course of the hospital stay (26). The resident research team also encouraged their peers to schedule timely follow-up within 7 days of discharge, and to review medications and symptom management with patients. In some areas, improvements were insufficient, and some variables being tracked worsened. Without resident tracking during the check phase of the process, appropriate corrective measures were much less likely to have been undertaken.

The STELR program is unusual in that it requires less financial resources compared to government funded programs such as Project RED and BOOST. In a community teaching hospital, house staff members can help maintain the program and reap the educational benefits they gain through peer-to-peer interaction and education and through collaboration with multidisciplinary teams to understand and utilize resources available within health care systems. An interdisciplinary program, such as STELR, is essentially a low budget solution to a high cost problem. Collaboration between physicians, nurses, case management, documentation specialists, social workers, and hospital administration was one of the most important factors in the success of STELR. Participation in the STELR program allowed the residents to gain an awareness of the larger context of the health care system, or systems-based practice, one of the fundamental competencies of their ACGME training requirements. More so, participation in STELR also provided them with a way to advocate for patients within the system (27).

To our knowledge, there is little published on the nature of resident involvement in hospital quality improvement programs. The STELR program can be easily replicated in other internal medicine residency programs. Involvement in the program was an invaluable part of resident training, fostering leadership in and ownership of every aspect of patient care. The resident co-authors (J.R., D.R.B., R.K., M.R., and J.V.) felt that their communication and interpersonal skills were strengthened by their interactions with the nursing and discharge planning staff. In addition, involvement in the program shed light on the intricacies of using system resources in healthcare. The STELR program could serve as a model for systems-based practice training in other residency programs in collaboration with hospital administration.

In the future, the STELR team hopes to expand the program to ensure patient follow up and track outpatient compliance. This could be especially useful in patients discharged to long-term nursing facilities, as they compose a large percentage of the population readmitted within 30 days. A nurse transition of care coordinator and increased collaboration with home health care agencies may also help increase outpatient compliance. This will allow residents to play a more active role in outpatient medical care in between doctor visits.

Despite ongoing patient tracking and program monitoring, it was difficult to quantify the resident-specific contribution to the STELR program. As with any multifaceted program, evaluation of the individual components is challenging. Another limitation was that there was no standard method of conducting the peer-to-peer education. Although clear objectives were addressed, real-time education was left to the discretion of the STELR resident team member. However, given the busy nature of resident physicians, it would have been difficult to implement a more standard approach, and utilizing patient-specific teachable moments is a more feasible plan of action in this environment. Finally, the retrospective and prospective patient data cannot be compared statistically because they represent two different groups of patients (CHF readmitted patients and CHF admissions). However, as this was a quality study, the retrospective portion was conducted to illuminate factors that may have contributed to readmission. Although, based on anecdotal evidence, the retrospective group was not sicker than the prospective group despite representing only readmitted patients (Table 1). This suggests that the patients in the prospective group did not have fewer readmissions simply because they were healthier than those in the retrospective portion.

## Conclusion

The efficacy of CHF readmission reduction programs is well documented. However, these programs can be costly to maintain and often require a substantial amount of human resources. The STELR program improved patient management and transition to home through accurate documentation, diagnosis, peer-to-peer education, patient education, and by securing outpatient home health care services and follow-up appointments. The benefit of program participation for the residents was twofold: Firstly, it provided a platform for the research team residents to serve as mentors to other residents. Secondly, it increased awareness of resources available within the health care system that not only have a great impact on patient care, but that are cost-effective and accessible to all patients. The inclusion of resident physicians in the STELR program demonstrates a cost-effective and reproducible initiative to reduce readmissions in coordination with multidisciplinary teams, spearheaded by the hospital administration. We believe the resources and costs associated with starting and maintaining the program was grossly outweighed by the improvement in patient outcomes, health care savings, and the overall enrichment of the residents’ education.
